# Incident Kaposi sarcoma during the expansion of antiretroviral therapy eligibility in Nigeria: a retrospective cohort study

**DOI:** 10.1186/s12885-023-11402-3

**Published:** 2023-09-21

**Authors:** Maxwell. O. Akanbi, Lucy. A. Bilaver, Chad Achenbach, Lisa. R. Hirschhorn, Adovich. S. Rivera, Orimisan. S. Adekolujo, Kehinde. U. A. Adekola, Olugbenga. A. Silas, Patricia. A. Agaba, Oche Agbaji, Nathan. Y. Shehu, Solomon. A. Sagay, Lifang Hou, Robert. L. Murphy

**Affiliations:** 1 Department of Hematology & Clinical Oncology, Michigan State University/ McLaren Greater Lansing, 2900 Collins Road, Michigan, 48910 USA; 2 Feinberg School of Medicine, Northwestern University, Chicago, IL USA; 3 Department of Medicine, McLaren Hospital, Flint, MI USA; 4 College of Medicine, University of Jos, Jos, Plateau State Nigeria

**Keywords:** HIV-associated Kaposi sarcoma, HIV-associated malignancies, Africa, Nigeria, Antiretroviral therapy, Epidemiology

## Abstract

**Introduction:**

The expansion of antiretroviral therapy (ART) eligibility could lead to earlier initiation of Human Immunodeficiency Virus (HIV) treatment and consequently reduce the risk of HIV-associated Kaposi Sarcoma (KS). We investigated the impact of changes in the Nigerian HIV treatment guidelines on KS incidence among adults enrolled in HIV care in Nigeria.

**Methods:**

We analyzed data of adults who enrolled for HIV care from January 2006 to December 2016 at one of Nigeria’s largest HIV treatment centers. Based on changes in HIV treatment guidelines, we classified 2006–2009 as the pre-expansion period and 2010–2016 as the post-expansion period. We used Kaplan Meier curves to compare the incidence of KS in the pre-expansion to the post-expansion period. We used Cox regression models to assess the hazard for incident KS between the two periods after adjusting for potential confounders.

**Results:**

Among 14,479 patients with HIV, the overall KS incidence was 2.35; 95% CI 2.01–2.74/1,000 person-years. The incidence of KS decreased from 2.53 to 1.58 per 1,000 person-years from 2006 to 2009 to 2010–2016. In models adjusting for age, sex, CD4-T cell count, and ART use, the risk for KS remained lower in 2010–2016 compared to 2006–2009. In analyses restricted to time on ART, there was no significant difference in KS incidence between HIV patients who enrolled in 2006–2009 and 2010–2016 after adjusting for age, sex, and CD4 T-cell count.

**Conclusion:**

The expansion of ART eligibility was associated with a reduced incidence of HIV-associated KS among adults initiating HIV care in Jos, Nigeria. The reduction was likely driven by earlier enrollment for HIV care and ART initiation.

**Supplementary Information:**

The online version contains supplementary material available at 10.1186/s12885-023-11402-3.

## Introduction

Antiretroviral therapy (ART) has significantly reduced the incidence of Kaposi sarcoma (KS) among people with Human Immunodeficiency Virus/ Acquired Immunodeficiency Syndrome (HIV/AIDS) globally [1–4]. Yet, in this modern ART era, KS remains one of the most common HIV-associated cancers in sub-Saharan Africa (SSA) [1, 5–7]. While ART and effective chemotherapy have dramatically improved survival in patients with HIV-associated KS in high-income countries, patients with HIV-associated KS continue to have poor treatment outcomes in low- and middle-income countries with limited access to oncologic services [8–11]. Cancer prevention should therefore be prioritized in SSA and other low-income settings [12].

In response to evolving evidence showing significant survival benefits of earlier ART initiation [13–16], Nigeria, an African country with the second-largest global HIV population, iteratively expanded access to ART. The most substantial changes to HIV treatment guidelines in Nigeria were made in 2010 and 2016 [14, 15]. In 2010, access to ART was extended to patients with CD4 T-cell count < 350 cells/mm [3] (as opposed to < 200 cells/mm3 or an AIDS-defining condition), and in 2016 all people with HIV were eligible for ART [14, 15]. While these guideline changes should facilitate ART initiation before the development of advanced HIV, which increases the risk for KS, a significant proportion of patients continued to present late to care [17]. Late initiation of HIV care may blunt the benefit expected from these guideline changes in the real world. To date, the impact of changes in HIV treatment guidelines on the risk for KS has not been systematically studied in Nigeria, despite its large HIV population and the endemicity for Human Herpes Virus type 8 (HHV8), the virus responsible for KS [18, 19]. We had previously investigated changes in KS prevalence among adults initiating HIV care and did not find a significant difference in KS prevalence at enrollment over four periods (2006–2008, 2009–2011, 2012–2014, and 2015–2017) [20]. In this study, utilizing data from the same clinic cohort, we investigate the impact of ART eligibility expansion on the incidence of KS among adults initiating HIV care.

## Methods

### Study design and setting

In this retrospective cohort study, we analyzed data of adults who initiated care at the Jos University Teaching Hospital (JUTH) HIV Clinic between January 1, 2006, and December 31, 2016, with follow-up until December 31, 2017. The JUTH HIV clinic, one of Nigeria’s oldest public HIV treatment centers, started enrolling patients for ART at the inception of Nigeria’s public ART program in 2002. The clinic received funding from the United States President’s Emergency Plan for AIDS Relief (PEPFAR) [21–23].

The clinic offered free ART to all eligible patients during the study period. ART eligibility was determined by Nigerian ART treatment guidelines [14, 15, 24]. From 2006 to 2009, the 2005 Nigerian HIV treatment guidelines were in effect. The guidelines recommended ART initiation in adults with CD4 T-cell count ≤ 200 cells/mm^2^ or World Health Organization (WHO) HIV stage 4 disease or WHO HIV stage 3 disease with CD4 T-cell count < 350 cells/mm^3^. In 2010 adults with CD4 T-cell count < 350 cells/mm^3,^ irrespective of WHO HIV stage or the presence of WHO HIV stage 3 or 4 HIV disease, became eligible for ART. In 2014, ART eligibility was extended to individuals with a CD4 T-cell count < 500 cells/mm^3^, HIV neuropathy, or coinfection with hepatitis B virus. By 2016, all patients with HIV, irrespective of CD4 T-cell count, were eligible for ART. The clinic monitored ART treatment response through scheduled CD4 T-cell count and HIV RNA measurement.

Our analytic population comprised ART-naive patients with HIV 18 years or older, free of KS at or within 30 days of clinic enrollment (We classified KS diagnosed within 30 days of initiation of care as prevalent KS).

Our study was approved by the Institutional Review Board (IRB) of the Jos University Teaching Hospital Research and Ethics Committee and was ruled exempt by the IRB of Northwestern University, Chicago.

### Data sources

We obtained de-identified, longitudinal data of patients who initiated HIV care from 2006 to 2016. The primary data source for this study was the JUTH HIV Clinic’s electronic health record system (EHRS). The EHRS was designed by the Harvard T.H-Chan School of Public Health using FileMaker Pro (FileMaker Pro, Santa Clara, CA ) and became operational in August 2004. The EHRS stored demographic, clinic, and laboratory records of all patients. To ensure data accuracy, the EHRS had built-in data error checks; additional checks were carried out by data managers each week.

### Study variables

Our primary outcome was incident KS, which we defined as the first diagnosis of KS after 30 days of enrollment for HIV care. KS diagnosis was either clinical or histologic. We computed follow-up time from initiation of HIV care to KS diagnosis, last clinic visit, or December 31, 2017, whichever came first.

The independent variable was the period of initiation of HIV care. Based on Nigeria’s ART eligibility expansion timeline [14, 15, 24], we classified 2006–2009 as the pre-expansion period and 2010–2016 as the post-expansion period. Covariates in our analyses include demographic characteristics and potential confounders. We included the following covariates: age (categorized as < 30, 30–50, and ≥ 50 years or as a continuous variable), sex (male or female), CD4 T-cell count (square root transformed) at baseline and ART initiation, HIV RNA at ART initiation (log-transformed), current ART use, and initial ART combination for patients who received ART (grouped as Protease Inhibitor + Nucleoside/Nucleotide reverse transcriptase inhibitor, Non-nucleoside reverse transcriptase inhibitor + Nucleoside/Nucleotide reverse transcriptase inhibitor, and Nucleoside/Nucleotide reverse transcriptase inhibitor only. For CD4 -T cell count at baseline or ART initiation, results within 15 days of the initiation of HIV care or ART were used. HIV RNA at ART initiation was defined as HIV RNA obtained within 30 days preceding ART initiation.

### Statistical analyses

We computed the crude KS incidence by dividing the number of KS cases in each period by the person-time follow-up (years), multiplied by 1,000. We used a Poisson model with generalized estimating equations to calculate incidence rate ratios. The model assessed the occurrence of KS for a given year while accounting for person-year contributed by an individual by the end of a calendar year.

We hypothesized that the expansion of ART eligibility led to a significant reduction in KS incidence. We used various Cox regression models to assess the hazard for incident KS between the pre-expansion (2006–2009) and post-expansion (2010–2016).We first ran bivariate analyses and then ran multivariable models adjusting for age, sex, and a marker of HIV severity (either CD4 T-cell count or HIV viral load). To adjust for the potential impact of ART on KS risk, we ran extended Cox models that adjusted for time-varying ART status and CD4 T-cell count or HIV viral load at baseline and at the time of ART initiation.

We conducted subgroup analyses that included only patients who initiated ART during follow-up. We ran descriptive statistics and KS incidence in this sub-population. We also ran a Cox model from the time of initiation of HIV care to ART initiation (Pre-ART period) and another model that started at ART initiation to the censoring date (Post-ART period).

### Sensitivity Analyses

For the primary analyses, we restricted our regression analyses to patients with complete datasets (*N* = 11,271). For the sensitivity analyses, we examined the effect of missing variables on the robustness of our analyses, using the multiple imputation method. Predictive mean matching was used to impute the missing data. Different analyses were done assuming missing at random or missing not at random. The details of the analyses are in the supplemental materials (Supplement Table S2-S4, Figure S2-S4).

Descriptive statistics and figures were run using Stata and survival models were run in R/RStudio. Multiple imputation was conducted using chain equations implemented using the mice package in 4 4.1.0/R studio.

## Results

### Participants

A total of 18,626 ART-naive patients aged 18 years or older initiated HIV care at the JUTH HIV clinic from January 1, 2006, to December 31, 2016. We excluded the following patients: 4,080 with less than 30 days of follow-up and 67 with more than 30 days of follow-up diagnosed with KS within 30 days of initiation of HIV care.

We analyzed data from 14,479 patients with 68,168 person-years of follow-up. The mean age of the population was 35 [9] years, and 67% (9,702) were female (Table 1). The median follow-up time was 3.8 years (IQR 1.1–8.3 years). Most patients (10,340; 71.41%) initiated HIV care pre-expansion. Patients who started HIV care in the post-expansion era were older, had higher CD4 T-cell counts, and presented at earlier stages of HIV disease (Table 1).

**Table 1:** Characteristics of adults who initiated HIV care in Jos, Nigeria (2006–2016)

		Year of initiation of HIV care		
	Overall (*n* = 14,479)	2006–2009 (*n* = 10,340)	2010–2016 (*n* = 4,139)	*P*-value
**Age, means (SD)**	35.0 (9.4)	34.9 (9.2)	35.3 (9.8)	0.01
**Age group, years, n (%)**				0.01
< 30	4,441 (30.7)	3,191(30.9)	1,250 (30.2)	
30–50	8,848 (61.1)	6,345 (61.4)	2,503(60.5)	
> 50	1,190 (8.2)	804 (7.8)	386(9.3)	
**Sex, n (%)**				0.80
Female	9,702 (67.0)	6,935 (67.1)	2,767 (66.9)	
Male	4,777 (33.0)	3,405 (32.9)	1,372 (33.2)	
**WHO Clinical Stage, n (%)**				< 0.01
1or 2	7,653 (52.9)	5,178 (50.1)	2,475 (59.8)	
3 or 4	4,029 (27.8)	3,003 (29.0)	1,026 (24.8)	
Missing	2,797 (19.3)	2,159 (20.9)	638 (15.4)	
**Baseline CD4 T-cell count, cells/mm** ^**3**^, **Median (IQR)**	175 (88–304)	167 (86–294)	191 (95–327)	< 0.01
**Baseline CD4 T-cell count, cells/mm** ^**3**^				< 0.01
< 200	7,745 (53.5)	5,614 (54.3)	2,131 (51.5)	
200–349	3,379 (23.3)	2,315 (22.4)	1,064 (25.7)	
350–499	1,452 (10.0)	972 (9.4)	480 (11.6)	
≥ 500	1,141 (7.9)	754 (7.3)	387 (9.4)	
Missing	762 (5.3)	685 (6.6)	685 (1.9)	
**ART > 30 days, n (%)**	12,650 (87.4)	8,790 (85.0)	3,860 (93.3)	< 0.01

*SD *Standard deviation, *WHO *World Health Organization, *HIV *Human Immunodeficiency Virus, *ART *Antiretroviral therapy

ART > 30 days: More than 30 days of ART use

Most patients (12,650; 87.4%) received ART. The median time from initiation of HIV care to the commencement of ART was 38 (IQR 29–144) days. This time interval progressively reduced over time from 52 (IQR 42–231) days in 2006 to 14 (IQR 13–18) days by 2016 (Supplemental Fig. 1).

### Antiretroviral therapy eligibility expansion and Kaposi sarcoma incidence

There were 160 incident cases of KS yielding a crude incidence rate of 2.35 (95% CI 2.01–2.74) per 1000 person-years. Table 2 shows the crude KS incidence by age group, sex, CD4 T-cell count at enrollment, and time since enrollment for HIV care. KS incidence was highest in the first six months of follow-up (14.54 per 1000 person-years) and progressively declined.

**Table 2:** Crude Kaposi sarcoma incidence in adults who initiated HIV care in Jos, Nigeria (2006–2016)

	Person-years	Number of Kaposi Sarcoma cases	Crude incidence rate (per 1000 person-years)	95% confidence interval
**Overall**	68121.0	160	2.35	2.01–2.74
**Age group, years**				
< 30	20412.4	52	2.55	1.94–3.34
30–50	42533.1	97	2.28	1.87–2.78
> 50	5176.0	11	2.13	1.17–3.84
**Sex, n (%)**				
Female	46260.6	89	1.92	1.56–2.37
Male	21860.9	71	3.25	2.57–4.10
**Baseline CD4-Tcell count, cells/mm** ^**3**^				
< 200	38001.0	105	2.76	2.28–3.35
200–349	17465.7	28	1.60	1.11–2.32
350–499	6928.8	16	2.31	1.41–3.77
≥ 500	4896.9	7	1.42	0.68–2.99
Missing	829.1	4	4.82	1.81–12.85
**Time since initiation of HIV care**				
< 6 months	6,751.5	86	12.73	10.31–15.74
6 months to < 1year	5,871.0	34	5.79	4.14–8.10
1 to < 1.5 years	5,306.8	13	2.45	1.42–4.22
1.5 to < 2 years	4890.6	15	3.07	1.85–5.09
≥ 2 years	4,5301.7	12	0.26	0.15–0.47

**Fig. 1:**
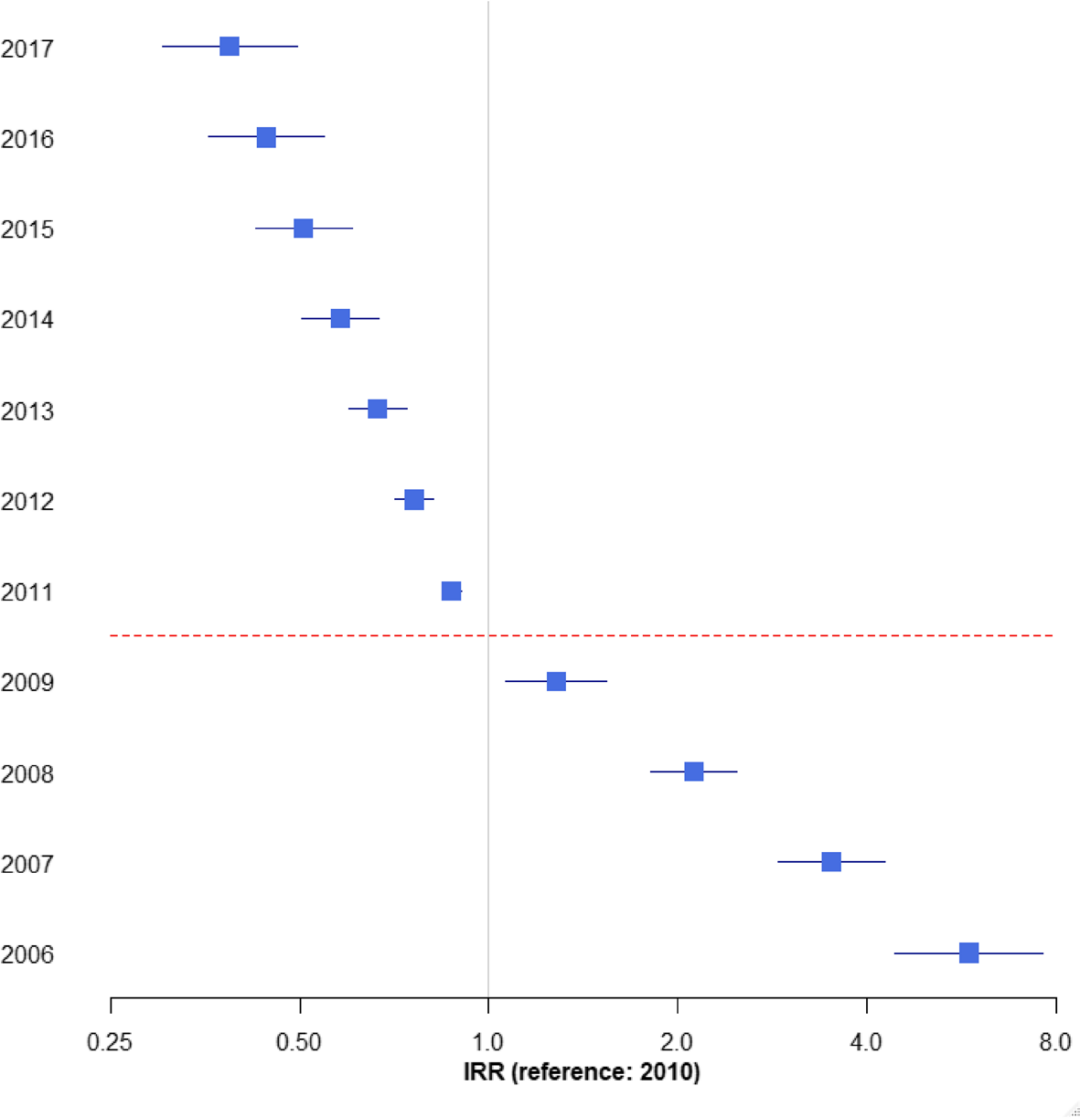
Kaposi sarcoma incidence rate ratios by calendar year. (Results obtained from unadjusted Poisson Model). IRR: Incidence rate ratio

From 2006 to 2009, we identified 139 incident cases of KS during a follow-up period of 54,851 person-years, with a crude incidence of 2.53 per 1,000 person-years. From 2010 to 2016, 21 incident cases of KS were recorded over 13,271 person-years, with a crude incidence of 1.58 per 1,000 person-years. Based on the calendar year, KS incidence progressively declined over time (Fig. 1). In Cox regression analyses adjusted for age and sex, the risk for KS was 37% lower in patients who enrolled for HIV care in 2010–2016 compared with 2006–2009 (HR 0.63, 95% CI 0.56–0.70, *p* < 0.01).

Table 3 shows the results of unadjusted and adjusted cox-regression models in patients with complete baseline for age, sex, CD4 T-cell count and HIV viral load. After adjusting for age, sex, ART use, and CD4 T-cell count, patients who initiated care in 2010–2016 still had a significantly lower risk of KS than 2006–2009. Other factors independently associated with an increased risk for KS were male sex, lower CD4 T-cell count, and use of antiretroviral therapy. HIV viral load was not associated with the risk for KS (Supplement Table S5 and S6). In the sensitivity analyses in which missing variables were imputed, results were similar to that of the main analyses (Missing at random: HR: 0.4, 95%CI: 0.25 to 0.64, *p* < 0.001; Missing not at Random: HR 0.4, 95% CI 0.25–0.64, p < 0.001), except for attenuation of the difference in the risk for KS in the time-varying models ( Supplement Table S2).

**Table 3:** Cox regression models of predictors of kaposi sarcoma among adults with HIV in Jos, Nigeria (2006–2016)

Characteristics	Model 1			Model 2			Model 3		
	*n* = 11,271, events = 125			*n* = 11,271, events = 125			*n* = 11,271, events = 125		
	HR	95%CI	p-value	HR	95%CI	p-value	HR	95%CI	*p*-value
Age, years	0.99	0.97 to 1.01	0.4929	1	0.97 to 1.02	0.68	1	0.97 to 1.02	0.6883
Sex									
Female	Reference			Reference			Reference		
Male	1.64	1.12 to 2.41	0.0071	1.65	1.11 to 2.44	0.0131	1.66	1.12 to 2.46	0.0117
CD4 T-cell count (square root) (Baseline for Model 1,, time-varying for others)	0.96	0.93 to 1	0.013	0.91	0.88 to 0.94	< 0.0001	0.91	0.88 to 0.95	< 0.0001
Enrollment period									
2006–2009	Reference			Reference			Reference		
2010–2016	0.31	0.17 to 0.57	0.0002	0.48	0.26 to 0.87	0.0165	0.48	0.26 to 0.87	0.0158
ART user (Yes)	-	-	-	0.17	0.1 to 0.27	< 0.0001	0.17	0.1 to 0.28	< 0.0001

Model 1: Model adjusted for age, sex, baseline CD4 T-cell count

Model 2: Time varying model adjusted for age, sex, baseline CD4 T-cell count, CD4 T-cell at start of ART, and ART use (CD4 T-cell count after ART was not updated for patients not on ART)

Model 3: Time varying model adjusted for age, sex, updated CD4 T-cell counts (from a year before ART commencement to 2 weeks after starting ART) and ART use. (CD4 T-cell count after ART was not updated for patients not on ART)

*ART *Antiretroviral therapy, *HIV *Human Immunodeficiency Virus, *HR *Hazard ratio

All analyses restricted to patients with complete baseline for age, sex, CD4 T-cell count and HIV viral load; *n*=11,271

### Kaposi sarcoma incidence among patients who received antiretroviral therapy

We included data from 12,650 patients who received ART in this subgroup analysis. We report the characteristics of patients in Supplemental Table 1; the majority were female (66.8%, 8,450), between the ages of 30–50 years (62%, 7,840), and had a CD4 T-cell count below 200 cells/mm^3^ at the time of ART initiation (60.5%, 7,646).

We identified 155 incident cases of KS over a follow-up period of 60,886 person-years, yielding an overall KS incidence of 1.02 (95% CI 0.79–1.31) per 1000 person-years. Time at risk was from initiation of HIV care. Although the incidence of KS was higher in patients who initiated HIV care in the pre-expansion compared to the post-expansion period (1.05 (95% CI 0.80–1.38) per 1000 person-years versus 0.89 (95% CI 0.49–1.61) cases per 1000 person-years) the difference was not statistically significant.

To elucidate the impact of ART use on KS risk, we conducted cox-regression analyses comparing the risk of KS in the pre-and post-expansion period before ART initiation and during ART use (Table 4). We restricted these analyses to patients who were prescribed ART and had complete dataset (*n* = 10,719). In the model which examined the risk for KS before ART initiation the risk for KS was 83% lower in patients who initiated HIV care in 2010–2016 compared to 2006–2009 (HR 0.17, 95% CI 0.06–0.48, *p* < 0.01). However, in analyses restricted to time on ART, the difference in KS risk between patients who initiated HIV care in 2010–2016 versus 2006–2009 narrowed (HR 0.48, 95% CI 0.23–1.01, *p* < 0.05).

**Table 4:** Multivariate cox regression models of predictors of kaposi sarcoma among adults with HIV in Jos, Nigeria based on use of antiretroviral therapy (2006–2016)

Characteristics	Before ART			After ART		
	*n* = 10,857, events = 69			*n* = 10,719, events = 55		
	HR	95% CI	p-value	HR	95%CI	p-value
**Age, years**	0.98	0.96 to 1.01	0.2691	1.01	0.98 to 1.04	0.6417
**Sex**						
Female	Reference			Reference		
Male	1.73	1.04 to 2.9	0.0357	1.59	0.9 to 2.81	0.1084
**CD4 T-cell count, cell/mm** ^**2**^ **(square root) (either baseline or at ART initiation)**	0.98	0.94 to 1.03	0.4124	0.97	0.91 to 1.02	0.2333
**Enrollment period**						
2006–2009	Reference			Reference		
2010–2016	0.17	0.06 to 0.48	0.0007	0.48	0.23 to 1.01	0.0545

*ART *Antiretroviral therapy, *HR *Hazard ratio

Analyses restricted to patients who received antiretroviral therapy, and had complete data on age, sex, baseline CD4 T-cell count (for ‘Before ART’ cohort) or CD4 T-cell count at ART initiation (for ‘After ART’ cohort),  and HIV viral load

## Discussion

Among patients who enrolled for HIV care in a large HIV treatment center in Nigeria from 2006 to 2016, the incidence of KS declined during ART eligibility expansion. The crude incidence of KS declined from 2.53 to 1.58 per 1,000 person-years from 2006 - 2009 to 2010-16. After adjusting for age, sex, and CD4-Tcell count, the risk for KS was 52% lower among patients who initiated HIV care from 2010 to 2016 compared to 2006–2009. When we restricted our analyses to the period patients were on ART, although the adjusted incidence of KS remained lower in the post-expansion compared with the pre-expansion period, this difference was no longer statistically significant.

Expanding access to ART has the potential to alter the epidemiology of HIV-associated malignancies in SSA. While several previous studies have evaluated the impact of ART on KS incidence [10, 25], the current study is one of the first to specifically investigate the effect of ART eligibility expansion on KS incidence in an African cohort. Analyses of data from over 280,000 adults with HIV in 22 countries showed that the expansion of ART eligibility resulted in earlier initiation of ART [25], which is similar to what we observed in our study. In another study in Zambia, expanding ART eligibility was associated with increased retention rates and timely ART initiation, particularly in newly eligible patients [26]. The current study contributes to the growing evidence of the positive impact of changes in ART treatment guidelines on HIV-associated cancer epidemiology in sub-Saharan Africa.

Adopting guidelines that stipulate earlier initiation of ART could contribute to a reduction in KS incidence through several mechanisms. One of the most important mechanisms may be preventing HIV-mediated CD4 T-cell depletion, which increases the risk for opportunistic infections and malignancies [1, 3, 18]. We found that patients in the post-expansion period initiated ART earlier, which may be in response to the changes in Nigeria’s HIV treatment guidelines [14, 27]. The median interval from initiation of HIV care to commencement of ART declined from 52 days in 2006 to 14 days in 2016. Also noteworthy was that patients in the post-expansion era commenced ART earlier despite having a higher CD4 T-cell count at the care initiation time.

Besides reducing the risk for KS through preventing CD4 T-cell depletion, early ART initiation also reduces HIV viral load. HIV viremia is hypothesized to contribute to increased KS risk by increasing the replication of HHV-8 and promoting angiogenesis and replication of spindle cells within the KS lesions [28–30]. However, we did not find an association between pre-ART HIV viral load and subsequent risk for KS. This was not surprising because ART rapidly reduces viral load within months of its initiation [31, 32]. So, HIV viremia may play a more significant role in the pathogenesis of HIV-associated KS in ART naïve individuals or during sub-optimal HIV treatment response. We were, however, unable to explore this further because we lacked longitudinal viral load data.

To better understand the impact of ART on KS risk, we restricted our analyses to time on ART. In these analyses, the risk for KS in the post-ART expansion era was 52% lower than in the pre–expansion after adjusting for age, sex, and CD4 T-cell count at the time of commencement of ART although this was not significantly significant. This result suggests that delay in ART initiation in the pre-expansion era, may have had led to residual increase in KS risk even among patients on ART. Delay in ART initiation has been associated with long term cancer risk [33]. In addition to expanding ART eligibility and enabling timely ART initiation, we observed a shift in the potency of ART. Triple-nucleoside/nucleotide combination therapy, which is less potent than NNRTI or PI-based therapy [34] was more often used in the pre-expansion period. We observed a shift towards the more potent PI-based therapy in the post-expansion period. Current guidelines recommend integrase strand transfer inhibitor (INSTI) class, and dolutegravir with two NRTI as preferred ART backbone as preferred first-line ART due to potency, high barrier to resistance, and better adverse effect profiles [35]. While no ART class has been shown to reduce the risk of KS specifically, more potent ART, with better safety profiles, will likely lead to more rapid CD4-T cell count recovery, faster decline in HIV viral load, and better HIV treatment adherence, which would contribute to further reductions in the incidence of HIV-associated KS.

Our findings have important implications for HIV-associated KS, particularly in SSA, where the incidence of HIV-associated KS remains high [1, 36, 37]. The high KS incidence in SSA is attributed to several factors, most importantly, a higher prevalence of HHV-8 [19] and late ART initiation [17]. ART became available in SSA in the early 2000s, about two decades after it became available in most high-income countries [38]. Efforts to improve ART coverage have yielded positive results in most countries in SSA, with some countries reporting ART coverage similar to those in high-income countries [39]. But the disruption of HIV services due to the COVID-19 epidemic raises concerns about the sustenance of HIV care, particularly in low-resource settings [40].

This study has some limitations, which should be considered while interpreting our findings. First, the data analyzed were from a single institution where patients had access to specialist physician care and may not represent the level of care in other facilities. Because the data was not primarily collected for research, some variables were missing, which may have reduced the power of our study, particularly in our subgroup analyses. Another limitation is that we did not have histologic confirmation of KS, which may have led to the misclassification of some cases. Early, asymptomatic KS may have been missed, which may underestimate the burden of the disease. Also, we could not determine the prevalence of HHV-8 infection in our study population. Current HIV treatment guidelines do not recommend routine testing for HHV-8 infection, and commercial assays for its diagnosis are unavailable in Nigeria. Lastly, our study was in the pre-COVID-19 era and did not account for the effects of COVID-19 on HIV epidemiology.

Despite these limitations, this study has several strengths. Our study is the largest on the incidence of HIV-associated KS in Nigeria, the country with the second-largest global HIV epidemic. Also, data for the analysis was obtained from an electronic health record system designed and maintained by the Harvard TH Chan School of Public Health, with mechanisms in place to ensure the high accuracy of data [23]. We also conducted robust statistical analyses, accounting for time-varying risk factors such as CD4 T-cell count and ART status.

In conclusion, expanding ART eligibility was associated with a decline in the incidence of HIV-associated KS. Late presentation, however, remained pervasive. Since the emergence of COVID-19, studies have reported significant disruptions in the HIV care continuum, which may have more pronounced effects in low-resource settings. These disruptions could aggravate late presentation and reduce ART compliance among patients in care. A multifaceted approach aimed at early HIV diagnosis, linkage to care, timely ART initiation, and consistent ART supply is needed to successfully eliminate HIV-associated KS in settings with the twin epidemic of HIV and HHV-8.

### Supplementary Information

**Additional file 1: Table S1. **Characteristics of adults who initiated ART in Jos, Nigeria (2006-2018). **Figure S1.** Box plot of time from enrollment in care to initiation of antiretroviral therapy in adults with HIV in Jos, Nigeria (2006-2016). **Table S2.** Cox regression of predictors of Kaposi Sarcoma using Multiply Imputed Data from adults with HIV in Jos, Nigeria (2006-2016) (*n*=14,479, events=160). **Table S3. **Missing Data Pattern.** Table S4.** MICE model and corresponding populations and variables. **Figure S2.** MICE Mode A (Analytical Models 1 to 3). **Figure S3.** MICE Model B (Analytical Model 4) and MICE Model C (Analytical Model 5). **Figure S4.** MICE Model D (Analytical Model 6). **Table S5.** Cox Regression models of predictors of Kaposi Sarcoma among adults with HIV in Jos Nigeria (2006-2016). T**able S6.** Multivariate cox regression models of predictors of Kaposi Sarcoma among adults with HIV in Jos, Nigeria based on use of antiretroviral therapy (2006-2016)

## Data Availability

All study data will be made available by the first author upon request.

## Abbreviations

AIDS: Acquired Immune Deficiency Syndrome
ART: Antiretroviral therapy
COVID-19: Coronavirus disease 2019
HIV: Human Immunodeficiency Virus
IQR: Interquartile range
KS: Kaposi Sarcoma
SD: Standard deviation
SSA: Sub-Saharan Africa
WHO: World Health Organization
