# Supplementary material for: Incident Kaposi sarcoma during the expansion of antiretroviral therapy eligibility in Nigeria: a retrospective cohort study
Source: BMC Cancer. 2023 Sep 21;23:890. doi: 10.1186/s12885-023-11402-3 (PMC10512500; doi:10.1186/s12885-023-11402-3)
Supplement: Supplementary file 1 — Additional file 1: Table S1. Characteristics of adults who initiated ART in Jos, Nigeria (2006-2018). Figure S1. Box plot of time from enrollment in care to initiation of antiretroviral therapy in adults with HIV in Jos, Nigeria (2006-2016). Table S2. Cox regression of predictors of Kaposi Sarcoma using Multiply Imputed Data from adults with HIV in Jos, Nigeria (2006-2016) (n=14,479, events=160). Table S3. Missing Data Pattern. Table S4. MICE model and corresponding populations and variables. Figure S2. MICE Mode A (Analytical Models 1 to 3). Figure S3. MICE Model B (Analytical Model 4) and MICE Model C (Analytical Model 5). Figure S4. MICE Model D (Analytical Model 6). Table S5. Cox Regression models of predictors of Kaposi Sarcoma among adults with HIV in Jos Nigeria (2006-2016). Table S6. Multivariate cox regression models of predictors of Kaposi Sarcoma among adults with HIV in Jos, Nigeria based on use of antiretroviral therapy (2006-2016) [file 12885_2023_11402_MOESM1_ESM.docx]

**Incident Kaposi Sarcoma during the expansion of antiretroviral therapy eligibility in Nigeria: A Retrospective Cohort Study**

Authors:

Maxwell. O. Akanbi, MD, Ph.D^1,2,3,4^

Lucy. A. Bilaver, Ph.D^2^

Chad Achenbach, MD, MPH^2^

Lisa. R. Hirschhorn, MD, MPH^2^

Adovich. S. Rivera, BS, MD^2^

Orimisan. S. Adekolujo, MD, MBA^3^

Kehinde. U. A. Adekola, MD, MS^2^

Olugbenga. A. Silas, MD, MS^4^

Patricia. A. Agaba, MD^4^

Oche Agbaji, MD, MPH^4^

Nathan. Y. Shehu, MD^4^

Solomon. A. Sagay, MD^4^

Lifang Hou, MD, Ph.D^2^

Robert. L. Murphy, MD^2^

Affiliations:

Michigan State University, Hematology, and Oncology, Greater Lansing, Michigan, United States

Northwestern University, Feinberg School of Medicine, Chicago, Illinois, United States

McLaren Hospital, Department of Medicine, Flint, Michigan, United States

University of Jos, College of Medicine, Jos, Plateau State, Nigeria

Corresponding Author:

Maxwell.O. Akanbi, MD, PhD

Department of Hematology & Clinical Oncology

Michigan State University/ McLaren Greater Lansing

2900 Collins Road, Lansing, Michigan. 48910

Email:Maxwell_akanbi@yahoo.com

**SUPPLEMENT 1.**

**Table S1:** Characteristics of adults who initiated ART in Jos, Nigeria (2006-2018)

|  | **Total** | **Period of initiation of HIV care** | | **p-value** |
| --- | --- | --- | --- | --- |
|  |  | **2006-2009** | **2010-2016** |  |
|  | **(N=12,650)** | **(n=8,790)** | **(n=3,860)** |  |
| **Sex, n (%)** |  |  |  | 0.79 |
| Female | 8,450 (66.80) | 5,865 (66.72) | 2,585 (66.97) |  |
| Male | 4,200 (33.20) | 2,925 (33.28) | 1,275 (33.03) |  |
| **Age group, years, n (%)** |  |  |  | 0.002 |
| <30 | 3,768 (29.79) | 2,632 (29.94) | 1,136 (29.43) |  |
| 30-50 | 7,840 (61.98) | 5,485 (62.40) | 2,355 (61.01) |  |
| ≥50 | 1,042 (8.24) | 673 (7.66) | 369 (9.56) |  |
| **CD4-Tcell count at ART initiation, cells/mm^3^, median (IQR)** | 138 (61 - 249) | 125 (56-226) | 156 (73-269) | <0.001 |
| **CD4-Tcell count at ART initiation, cells/mm^3^, n (%)** |  |  |  | <0.001 |
| <200 | 7,646 (60.50) | 5,524 (62.93) | 2,122 (54.99) |  |
| 200-349 | 3,129 (24.76) | 2,113 ( 24.07) | 1,016 (26.33) |  |
| 350-499 | 1,146 (9.07) | 718 (8.18) | 428 (11.09) |  |
| ≥500 | 716 (5.67) | 423 (4.82) | 293 (7.59) |  |
| **WHO. Clinical Stage, n (%)** |  |  |  | <0.001 |
| 1 & 2 | 7,630 (60.32) | 5,158 (58.68) | 2,472 (64.04) |  |
| 3 & 4 | 4,019 (31.77) | 2,995 (34.07) | 1,024 (26.53) |  |
| Missing | 1,001(7.91) | 637 (7.25) | 364 (9.43) |  |
| **HIV RNA at ART initiation, copies/ml, n (%)** |  |  |  | <0.001 |
| <1,000 | 6,785 (53.64) | 4,755 (54.10) | 2,030 (52.59) |  |
| 1,000-9,999 | 1,521 (12.02) | 1,249 (14.21) | 272 (7.05) |  |
| 10,000-99,999 | 1,499 (11.85) | 1,149 (13.07) | 350 (9.07) |  |
| ≥100,000 | 1,051(8.31) | 780 (8.87) | 271 (7.02) |  |
| Missing | 1,794 (14.18) | 857 (9.75) | 937 (24.27) |  |
| **Class of ART, n (%)** |  |  |  | <0.001 |
| NNRTI+ NRTI/NtRTI | 12,311 (97.34) | 8,547 (97.27) | 3,764 (97.51) |  |
| Protease Inhibitor+ NRTI/NtRTI | 128 (1.01) | 44 (0.50) | 84 (2.18) |  |
| NRTI/NtRTI only (Triple Nukes) | 208 (1.64) | 196 (2.23) | 12 (0.31) |  |

ART: Antiretroviral therapy; IQR: Interquartile range; HIV: Human Immunodeficiency Virus; KS: Kaposi Sarcoma; NRTI: Nucleoside reverse transcriptase inhibitors, NNRTI: Non-nucleoside reverse transcriptase inhibitors, NRTI: Nucleoside reverse NtRTI: Nucleotide reverse-transcriptase inhibitors; WHO: World Health Organization


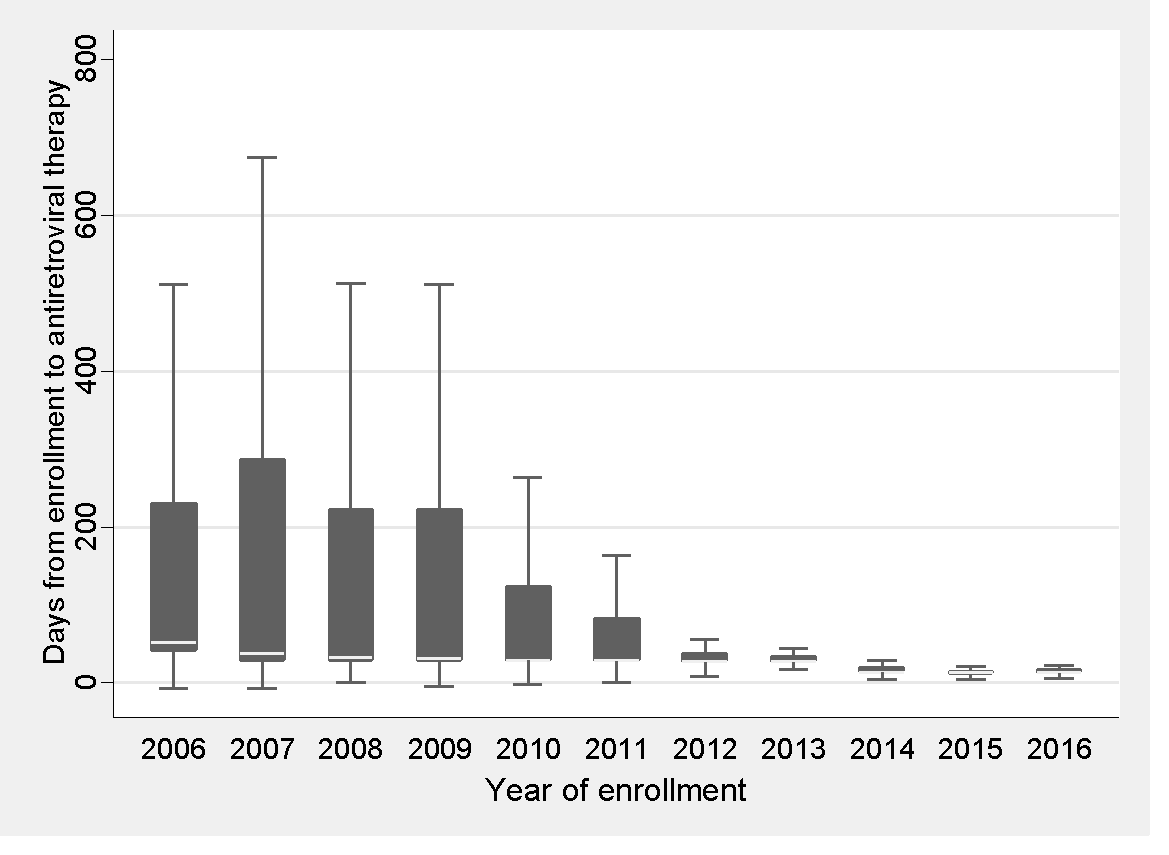


**Figure S1**: Box plot of time from enrollment in care to initiation of antiretroviral therapy in adults with HIV in Jos, Nigeria (2006-2016)

**Table S2:** Cox regression of predictors of Kaposi Sarcoma using Multiply Imputed Data from adults with HIV in Jos, Nigeria (2006-2016) (n=14,479, events=160)

1. Missing at Random

| Characteristics | Model 1 | | | Model 2 | | | Model 3 | | |
| --- | --- | --- | --- | --- | --- | --- | --- | --- | --- |
|  | HR | 95%CI | p-value | HR | 95%CI | p-value | HR | 95%CI | p-value |
| Age, years | 0.98 | 0.97-1 | 0.0944 | 0.99 | 0.97-1.01 | 0.1547 | 0.99 | 0.97-1.01 | 0.1587 |
| Sex |  |  |  |  |  |  |  |  |  |
| Female | Reference | |  | Reference | |  | Reference | |  |
| Male | 1.7 | 1.21-2.39 | 0.0022 | 1.65 | 1.18-2.31 | 0.0039 | 1.66 | 1.18-2.32 | 0.0035 |
| CD4 T-cell count (square root) (Baseline for original, time-varying for others) | 0.95 | 0.93-0.98 | 0.0016 | 0.91 | 0.88-0.94 | <0.0001 | 0.91 | 0.88-0.94 | <0.0001 |
| Enrollment period |  |  |  |  |  |  |  |  |  |
| 2006-2009 | Reference | |  | Reference | |  | Reference | |  |
| 2010-2016 | 0.4 | 0.25-0.64 | 0.0001 | 0.62 | 0.39-1 | 0.0492 | 0.62 | 0.38-1 | 0.0488 |
| ART user (Yes) | - | - | - | 0.19 | 0.12-0.28 | <0.0001 | 0.19 | 0.12-0.28 | <0.0001 |

1. Missing Not at Random

| Characteristics | Model 1 | | | Model 2 | | | Model 3 | | |
| --- | --- | --- | --- | --- | --- | --- | --- | --- | --- |
|  | HR | 95%CI | p-value | HR | 95%CI | p-value | HR | 95%CI | p-value |
| Age, years | 0.98 | 0.97-1 | 0.0949 | 0.99 | 0.97-1.01 | 0.1538 | 0.99 | 0.97-1.01 | 0.1583 |
| Sex |  |  |  |  |  |  |  |  |  |
| Female | Reference | |  | Reference | |  | Reference | |  |
| Male | 1.7 | 1.21-2.38 | 0.0023 | 1.64 | 1.17-2.3 | 0.0041 | 1.65 | 1.18-2.32 | 0.0037 |
| CD4 T-cell count (square root) (Baseline for original, time-varying for others) | 0.95 | 0.93-0.98 | 0.0019 | 0.91 | 0.88-0.94 | <0.0001 | 0.91 | 0.89-0.94 | <0.0001 |
| Enrollment period |  |  |  |  |  |  |  |  |  |
| 2006-2009 | Reference | |  | Reference | |  | Reference | |  |
| 2010-2016 | 0.4 | 0.25-0.64 | 0.0002 | 0.62 | 0.38-1 | 0.0488 | 0.62 | 0.38-1 | 0.0483 |
| ART user (Yes) | - | - | - | 0.2 | 0.13-0.29 | <0.0001 | 0.2 | 0.13-0.29 | <0.0001 |

Model 1: Model adjusted for age, sex, baseline CD4 T-cell count

Model 2: Time varying model adjusted for age, sex, baseline CD4 T-cell count, CD4 T-cell at start of ART, and ART use

Model 3: Time varying model adjusted for age, sex, updated CD4 T-cell counts (from a year before ART commencement to 2 weeks after starting ART) and ART use

ART: Antiretroviral therapy; HIV: Human Immunodeficiency Virus; HR: Hazard ratio

**Supplemental Methods and Results for multiple imputation**

Multiple imputation was conducted using multiple imputation using chained equations implemented using the mice package in R 4.1.0/RStudio.

1. Amount of Missing data

Most of the missing data were related to the CD4 and viral load variables. The degree of missingness in the data is shown in Table S4.

**Table S3. Missing Data Pattern**

| Pattern | n | % of data | Time to KS/Censoring | Had KS | sex | age | cohort group | ART initiation | CD4 at baseline (square root) | CD4 at ART (square root) | VL at baseline (log) | VL at ART (log) | # Variables missing in pattern |
| --- | --- | --- | --- | --- | --- | --- | --- | --- | --- | --- | --- | --- | --- |
| 1 | 2038 | 14% | Y | Y | Y | Y | Y | Y | Y | Y | Y | Y | 0 |
| 2 | 8746 | 60% | Y | Y | Y | Y | Y | Y | Y | Y | Y | N | 1 |
| 3 | 1729 | 12% | Y | Y | Y | Y | Y | Y | Y | Y | N | N | 2 |
| 4 | 4 | 0% | Y | Y | Y | Y | Y | Y | Y | N | Y | Y | 1 |
| 5 | 483 | 3% | Y | Y | Y | Y | Y | Y | Y | N | Y | N | 2 |
| 6 | 717 | 5% | Y | Y | Y | Y | Y | Y | Y | N | N | N | 3 |
| 7 | 1 | 0% | Y | Y | Y | Y | Y | Y | N | N | Y | N | 3 |
| 8 | 761 | 5% | Y | Y | Y | Y | Y | Y | N | N | N | N | 4 |
| total missing per variable | count | | 0 | 0 | 0 | 0 | 0 | 0 | 762 | 1966 | 3207 | 12437 |  |
|  | percent | | 0% | 0% | 0% | 0% | 0% | 0% | 5% | 14% | 22% | 86% |  |

Note: Y = available, N = not available or missing. Pattern 1 shows # of people with data for all the variables. Patterns 2 to 8 are the different types of missing data. For example, Pattern 2 comprises 60% of the people in the data and these are people with missing viral load at ART initiation.

1. Reasons for missingness

Missing data could be from a variety of reasons including loss to follow-up and testing practices during the time of the study. Due to cost constraints, HIV viral load testing was not routinely done as standard of care enrolling in care or commencing ART. Also, patients who met criteria for ART irrespective of CD4 count (those with AIDS defining conditions) may have commenced ART without a current CD4 cell count.

1. Method and imputation models

Four sets of models were used: Mice model A for Analytical model 1 to 3, Mice Model B for Analytical model 4, Mice model C for Analytical model 5, and Mice model D for Analytical model 6. For each model, there were 30 iterations and 70 imputed datasets. The population and variables included in the imputation models as described in Table S4.

Predictive mean matching was used to impute the missing variables. We noted that the imputed CD4 for model 1 to 3 seem to be systematically deviated from the original dataset. To assess the impact of this deviation, we conducted an additional analysis which assumes missing not at random where the imputed CD4 value was adjusted (reduced by 70% for the baseline CD4 and 80% for the CD4 at ART initiation) so that the peak of the original data and imputed datasets are aligned (see density plots below). We did not do this analysis for MICE Models C and D due to alignment of data distribution of imputed CD4 to the original data.

**Table S4. MICE model and corresponding populations and variables**

| MICE model | Analytical Model | Population | Variables |
| --- | --- | --- | --- |
| A | 1, 2, 3 | All eligible | Outcome, Time to Event, Nelson Alen estimator, Sex, Age, CD4 at baseline (square root), CD4 at ART initiation (square root), Viral Load at baseline (log), Viral Load at ART initiation (log) |
| B | 4 | All eligible | Outcome, Time to Event, Nelson Alen estimator, Sex, Age, CD4 at baseline (square root), Viral Load at baseline (log) |
| C | 5 | Eligible who started ART during observation | Outcome, Time to Event, Nelson Alen estimator, Sex, Age, CD4 at baseline (square root), Viral Load at baseline (log) |
| D | 6 | Eligible who started ART during observation | Outcome, Time to Event (time zero is ART start date), Nelson Alen estimator, Sex, Age, CD4 at baseline (square root), CD4 at ART initiation (square root), Viral Load at baseline (log), Viral Load at ART initiation (log) |

1. Diagnostic Density plots

**Figure S2: MICE Mode A (Analytical Models 1 to 3)**

| Assumes Missing at Random | Assumes Missing Not at Random |
| --- | --- |
| 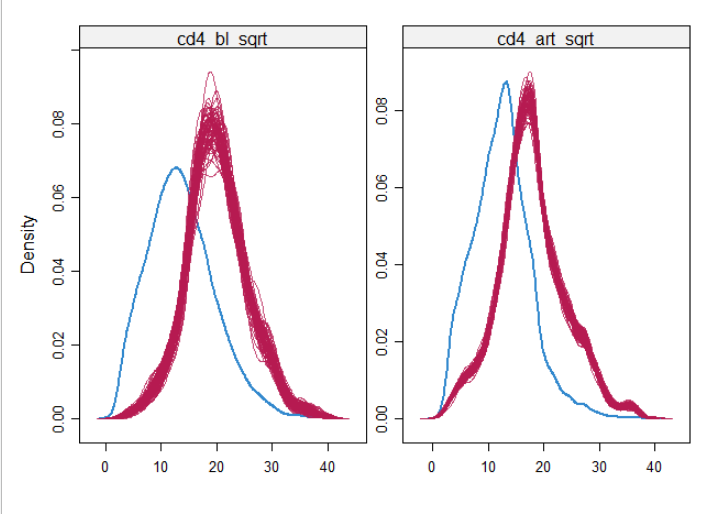 | 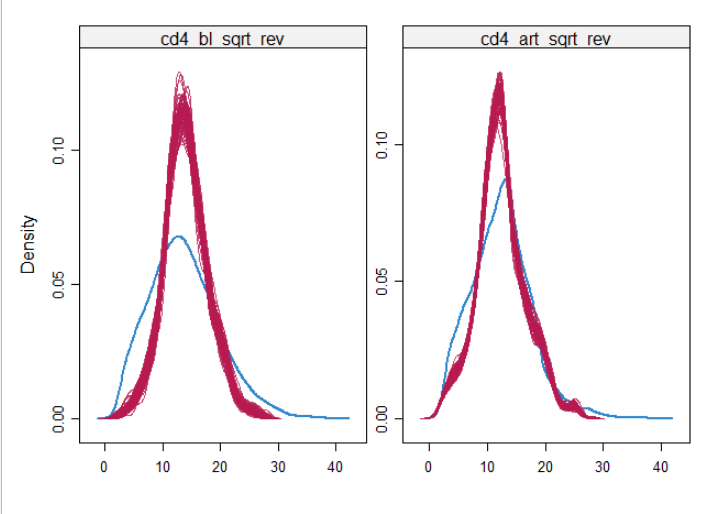 |

**Figure S3: MICE Model B (Analytical Model 4) and MICE Model C (Analytical Model 5)**

| CD4 at Baseline (both initiators and non-initiators of ART) | CD4 at baseline (only those who initiated ART) |
| --- | --- |
| 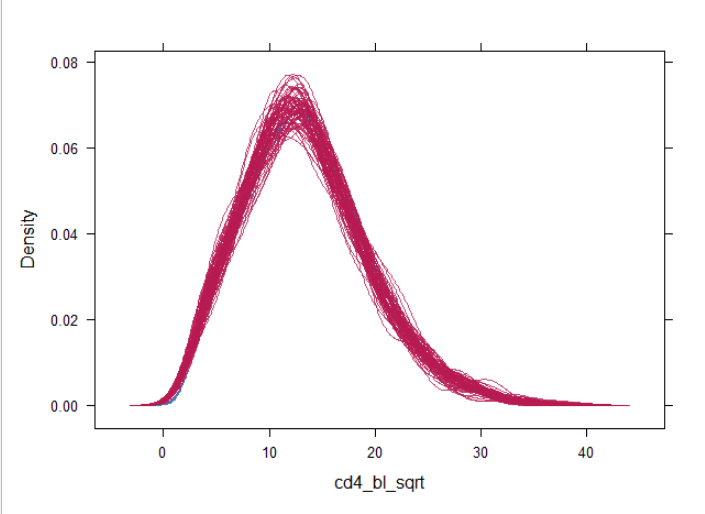 | 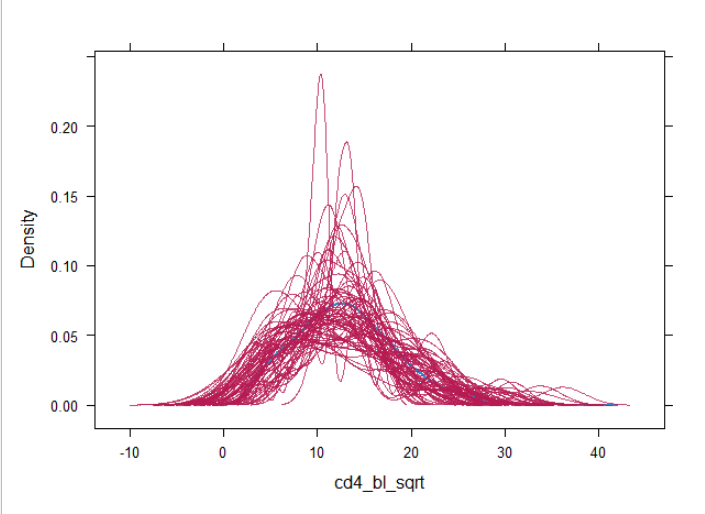 |

**Figure S4: MICE Model D (Analytical Model 6)**


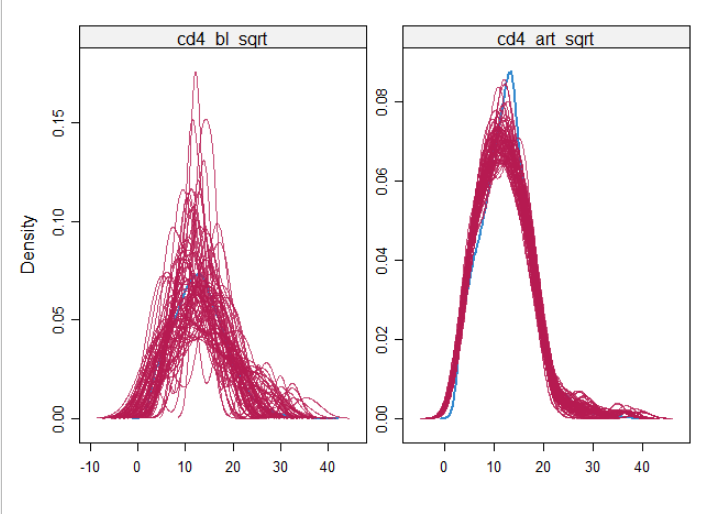


For all MICE models (Figures S2, S3, and S4): Original data is ‘Red’, imputed data is ‘Blue’.

**Table S5: Cox Regression models of predictors of Kaposi Sarcoma among adults with HIV in Jos Nigeria (2006-2016)**

| Characteristics | Original model | | | Time varying model adding ART status and Viral load at start of treatment | | |
| --- | --- | --- | --- | --- | --- | --- |
|  |  |  |  | Fully adjusted (Viral load at baseline) | | |
|  | n = 11,271, events = 125 | | | n = 11,271, events = 125 | | |
|  | HR | 95%CI | p-value | HR | 95%CI | p-value |
| Sex, Male | 1.72 | 1.18 to 2.51 | 0.0029 | 1.9 | 1.29 to 2.8 | 0.0013 |
| Age (Years) | 1 | 0.98 to 1.02 | 0.5907 | 1 | 0.98 to 1.02 | 0.7452 |
| Viral load (log) (Baseline for original, time-varying for others) | 1.06 | 1 to 1.13 | 0.0837 | 0.98 | 0.91 to 1.06 | 0.6279 |
| Enrollment Period |  |  |  |  |  |  |
| 2006-2009 | Reference | | |  |  |  |
| 2010-2015 | 0.32 | 0.18 to 0.58 | 0.0002 | 0.4 | 0.22 to 0.73 | 0.0027 |
| ART user |  |  |  | 0.26 | 0.17 to 0.4 | <0.0001 |

Analyses restricted to patients with complete results of baseline CD4 T-cell count and HIV RNA.

Table S6: Multivariate cox regression models of predictors of Kaposi Sarcoma among adults with HIV in Jos, Nigeria based on use of antiretroviral therapy (2006-2016)

| Characteristics | Before ART (exclude never user) | | | After ART | | |
| --- | --- | --- | --- | --- | --- | --- |
|  | n = 10,857, events = 69 | | | n = 2,032, events = 7 | | |
|  | HR | 95%CI | p-value | HR | 95%CI | p-value |
| Sex, Male | 1.76 | 1.06 to 2.92 | 0.0303 | 2.73 | 0.55 to 13.5 | 0.2171 |
| Age (Years) | 0.99 | 0.96 to 1.01 | 0.3192 | 1.01 | 0.93 to 1.09 | 0.8889 |
| HIV RNA (log) time-varying. | 1.09 | 1.01 to 1.19 | 0.0301 | 1.31 | 0.87 to 1.96 | 0.1981 |
| Enrollment Period |  |  |  |  |  |  |
| 2006-2009 | Reference | | | Reference | | |
| 2010-2015 | 0.19 | 0.07 to 0.51 | 0.0011 | 0.9 | 0.11 to 7.47 | 0.9203 |
| ART: Antiretroviral Therapy; HR: Hazard ratio.  Analyses restricted to patients who received antiretroviral therapy | | | | | | |
